# Barriers and facilitators influencing midwives’ implementation of South Africa’s maternal care guidelines in postnatal health: a scoping review

**DOI:** 10.1017/S1463423625000015

**Published:** 2025-02-28

**Authors:** Ngozichika Okeke, Roinah Ngunyulu

**Affiliations:** Department of Nursing, University of Johannesburg, Johannesburg, South Africa

**Keywords:** midwives roles, postnatal health care services, postnatal phase, South Africa maternal care guidelines

## Abstract

**Introduction:**

The implementation of South Africa’s maternal care guidelines is still subpar, especially during the postnatal periods, despite midwives playing a key part in postnatal care for women and their newborns. This article aimed to pinpoint the obstacles to and enablers of midwives’ roles in putting South Africa’s maternal care recommendations for postnatal health into practice.

**Method:**

A scoping review was conducted following Arksey and O’Malley method. Systematic searches were conducted using the PsycINFO, Nursing and Allied Health (CINAHL), PubMed, EBSCOhost web, and Google Scholar. The screening was guided by the inclusion and exclusion criteria. Data were analyzed using the Braun and Clarke method for thematic content analysis and included 22 articles. The quality of included studies was determined by Mixed Method Appraisal Tool and these were reported in accordance with Preferred Reporting Items for Systematic Reviews and Meta-analysis for Scoping Review.

**Results:**

There is a gap between inadequate postnatal care services provision and suboptimal implementation of maternal recommendations. Owing to a lack of basic knowledge about the guidelines, an absence of midwives in the maternity units, inadequate facilities and resources, a lack of drive and support, inadequate training of midwives in critical competencies, and poor information sharing and communication. Maintaining qualified midwives in the maternity units and providing them with training to increase their capacity, knowledge, and competencies on the guidelines’ critical information for managing postnatal complications and providing high-quality care to women and their babies is necessary to effectively implement the recommendations.

**Conclusion:**

The relative success in implementing maternal care guidelines in South Africa lies in the contextual consideration of these factors for the development of intersectoral healthcare packages, strengthening health system collaborations, and stakeholder partnerships to ameliorate maternal and newborn morbidity and mortality.

## Background

The postnatal period is important for a woman’s health and well-being as well as that of her child, and ineffective postnatal care could result in maternal and infant deaths (World Health Organization, 2018). Women and infants are at risk for postnatal problems like bleeding, hypertension, low birth weight, sepsis, and death in the postnatal period; therefore, the midwives are pivotal in the prevention of such issues and promotion of health after birth (World Health Organization, 2018; Ngunyulu *et al.*, 2020).

The American College of Obstetrics and Gynaecology (ACOG) (2018:1) defines the ‘postnatal period’ as an evolving process that ensures a woman and her infant’s optimal health and well-being (American College of Obstetricians and Gynaecology, 2018). This implies that the postnatal period is a continuous process rather than a one-time event during which support and services should be provided to meet the needs of the mother and infant (ACOG, 2018). ACOG recommends that postnatal care begins within the first three weeks of pregnancy and that the initial assessment be followed up with ongoing medical supervision as needed, with a complete postnatal assessment completed twelve weeks after birth. Maternal, physical, emotional, and social well-being, child care, breastfeeding, sexuality, contraception, and health maintenance and promotion are among the services provided. Patients with chronic illnesses like; diabetes, obesity, hypertension, and mood disorders should also receive appropriate counsel regarding postpartum appointments(American College of Obstetricians and Gynaecology, 2018).

A significant public health challenge is the postpartum maternal and infant mortality rate (World Health Organization (WHO), 2018). Many may suffer from severe health problems and long-term disabilities irrespective of whether the mom or child survives (Abebo and Tesfaye, 2018; Bradshaw and Dorrington, 2017). According to the World Health Organization (2018) and the National Committee for Confidential Enquiry into Maternal Deaths (NCCEMD) (2018), efforts to reduce preventable maternal and infant deaths have been ongoing globally.

Maternal and infant deaths have steadily decreased, according to studies from high- and middle-income nations; however, Africa is still making minimal progress (Benova, *et al.*, 2019). To lower morbidity and mortality rates, there is still much work to be done, especially in terms of developing policies aimed at enhancing midwives’ role in providing post-natal care to women and their babies, particularly during the postnatal periods (Moodley, Fawcus, & Pattison, 2018; Mulaudzi *et al.*, 2017).

There have been studies on the effects of maternal and infant mortality in the postnatal period. According to statistics, about 1.2 million African babies lose their lives within the first few days. In addition, a percentage of the roughly 850,000 African babies die during the postnatal period as a result of poor postnatal care. (World Health Organization, 2018; National Committee for Confidential Enquiry into Maternal Deaths, 2019; Abebo, and Tesfaye, 2018). Of the 125,000 maternal deaths that occurred during the postnatal period in 2017–2019, 50% of the deaths occurred within the first few hours following birth and half of all maternal deaths occurred within the first seven days during the post-partum period (WHO, 2018:4; Dorrington, Bradshaw & Laubscher, 2019; Chhetri, Shah, & Rajbansh, 2020).

Data from the most recent triennial and interim Saving Mothers Reports 2018 (S.M.R.s) highlights that child fatality rates (C.F.R.s) due to haemorrhage have increased from 15% to 40% while deaths, due to the complications of postpartum hypertension have increased from 10% to 55% in South Africa (Saving Mothers Report, 2018).

The SMR (NDoH, 2018:106) reports that there were 648 maternal deaths in SA from direct causes, of which 218 were attributable to high blood pressure and 181 to obstetric haemorrhage. According to the South African National Department of Health, (2018), National Committee for Confidential Enquiry into Maternal Deaths, (2019), Moodley, Fawcus, & Pattison, (2018), there may still be pronounced disparities in the ratios and implementation strategies for postnatal care for women and infants.

The following are some of the guidelines that have been developed to help save lives and enhance the health of pregnant women and their unborn children: Guidelines for postnatal care from the National Institute for Health Care Excellence (2019); recommendations for maternal care from the American College of Obstetrics and Gynecology (ACOG) (American College of Obstetricians and Gynecologists, 2018). The National Consolidated Guidelines for the Prevention of Mother-to-Child Transmission of HIV (2019), the WHO Guidelines on Routine Post-partum Care (2018); the WHO Guidelines on Postnatal Care for Mothers and Neonates (2018), and the South African Maternal Care Guidelines (2015). Only a few studies, however (Sayinzoga *et al.*, 2018;Siseho *et al.*, 2022) have looked at the extent to which these guidelines and publications have been followed.

A guideline for the role of midwives in implementing effective postnatal care to mothers and newborns is provided by the South African maternal care guidelines (South African Department of Health, 2017). In this recommendation, midwives’ roles in the care and management of women during the postpartum period are evaluated along with health system factors. The maternal care guidelines for postnatal care looked at aspects like midwives’ role in the postpartum care and management of women and their infants, practices like patient discharge within six hours of normal vertex delivery in acceptable conditions based on the discretion of the health provider, and follow up care (South African National Department of Health, 2018). The managing of episiotomies, taking vital signs, and midwives’ understanding, expertise, and capabilities during postnatal emergencies are also included in this list of contributing factors. These elements might have changed over time, but they have not been sufficiently explored due to difficulties in putting these guidelines into practice.

Examining maternal care recommendations for postnatal care reveals some advancement in the treatment provided to HIV-positive mothers to prevent mother-to-child transmission of the virus. Improved breastfeeding counselling services, EPI (Expanded Programme on Immunization) recommendations, HIV testing, and antiretroviral therapy are primarily responsible for the sudden decrease in mortality. Post-partum haemorrhage and post-partum hypertension, decreased the direct cause of maternal deaths (South African National Department of Health, 2018; National Committee for Confidential Enquiry into Maternal Deaths, 2019; Moodley *et al.*, 2018). Therefore, effective implementation of these recommendations in terms of care, referral networks, and personnel may help address this issue.

Additionally, little is known about the advantages of applying the South African Maternal Guidelines in terms of postnatal care (Saving Mothers Report, 2018). The survey revealed that despite efforts to implement recommendations for maternal care. There are frequently no obvious signs of success (Demographic Health Survey, 2017). Some surveys (Tessema *et al.*, 2020; Abota, Tadele, & Atenafu, 2018; Chungu, Makasa, & Chola, 2018) assume that most women who give birth in hospitals automatically receive postnatal care. According to the analysis of the Demographic and Health Surveys conducted in 2017 (Demographic Health Survey), only a small percentage of women, receive adequate postpartum care.

Also, instances of timely treatment and proper follow-up, particularly during the postnatal periods are poorly reported (Saving Mothers Report, 2017). Wherever patients enter the health system, their care should be managed appropriately as part of the continuity of care. The South African National Department of Health (2018) and Saving Mothers Report (2018) state that 25% of preventable maternal deaths are frequently attributed to a shortage of skilled midwives offering a continuum of care in the postnatal periods. According to the South African Maternal Care Guidelines, midwives are crucial in providing mothers and newborns with high-quality postpartum care (Department of Health, 2015).

Midwives are significant personnel in the health care system in the context of postnatal health. According to the maternal care recommendations (National Committee for Confidential Enquiry into Maternal Deaths, 2019; South African Department of Health, 2015) they offer postnatal care services to mothers and babies. However, some barriers affect how well-informed they are about maternal care guidelines, how well-retained midwives are in maternal care units, how well-equipped and oriented midwives are, how motivated midwives are, how well resources are developed, and how well-funded they are (National Committee for Confidential Enquiry into Maternal Deaths (NCCEMD), 2018; Mulaudzi, *et al.*, 2017).

Thus, it is essential to support midwives’ roles in developing and putting into action suitable measures. The same goes for maternal care guidelines: more people need to know the basics, make sure that the guidelines are followed in their roles during the postnatal period, orient and train midwives about the guidelines, expand the number of midwives and midwifery education programmes, encourage midwives to take effective action during the postnatal period, and build capacity and preparedness during emergencies to fill in gaps in the service delivery systems (Bingham *et al.*, 2018; Walker *et al.*, 2019; Ngunyulu *et al.*, 2018).

When competent midwives provide effective postnatal care services, there is a decrease in maternal and newborn mortality rates as well as positive health outcomes (Walker *et al.*, 2019). Promoting midwives’ roles in the provision of high-quality postnatal care thus depends on a supportive environment, the availability of resources and equipment, the expansion of human resources through hiring and retention of more midwives, the development of skills, ongoing education, effective leadership, enabling policies and collaboration with stakeholders, as well as effective referral system (Jordan *et al.*, 2018; Kumbiley *et al.*, 2021).

Therefore to implement the maternal care guidelines and make it easier for patients to access postnatal care services, the review set out to fill this gap in the literature by examining and describing the barriers and facilitators that influence implementation of the maternal guidelines on postnatal care in practice. This review aims to address the current gap in knowledge regarding the barriers and facilitators that influence midwives, in putting the maternal guidelines on postnatal care into practice.

## Methods

### Study design

The initial idea for the review and design was led by NO with support from RN. This methodology is especially suitable in cases where the primary sources and forms of existing evidence are intricate or have not undergone a thorough examination previously. The 5-step methodological framework presented by Arksey and O’Malley (2005) served as the basis for this review. These steps are as follows: [1] formulating the research question; [2] finding pertinent studies; [3] selecting and evaluating studies; [4] charting the data; and [5] compiling, analyzing, and disseminating the findings. The scoping review was then reported in accordance with the Preferred Reporting Items for Systemic Reviews and Meta-Analyses for Scoping Review (PRISMA-ScR) (Moher *et al.*, 2015). PRISMA-ScR specifically targets scoping reviews and offers detailed guidance on the ScR protocols. It is tailored to enhance the quality and clarity of scoping reviews, particularly in exploring interventions across various field, including medical, quantitative, mixed, and qualitative research to ensure transparency (Miranda *et al.*, 2023).

#### Stage 1: Identifying the research questions

Arksey and O’Malley (2005) suggest that it is important to formulate research questions, as it guides the review process. The following research questions were formulated after carefully reading through the topic.

1. what are the barriers that influence the midwives’ implementation of maternal care guidelines in postnatal health?
2. what are the facilitators that influence the midwives’ implementation of maternal care guidelines in postnatal health?

#### Stage 2: Identifying relevant studies

A strategic search process was adopted to capture a wide range of relevant studies on factors influencing midwives’ implementation of maternal recommendations in postnatal health using the following electronic databases: The University of Johannesburg database, Medline, PubMed, EBSCOhost, PsycINFO, Nursing and Allied Health (CINAHL plus), and Google Scholar. The bibliographies of key papers obtained from the databases were screened to identify further relevant papers. Searches of databases were pre-determined to identify all available evidence. Retrieved records were downloaded and stored in an information form to support management and screening. Articles were searched through the ‘Cited by’ search as well as citations included the reference lists included articles. The search terms included; ‘maternal care guidelines implementation globally’. Maternal guidelines implementation in sub-Saharan Africa, ‘barriers to implementation of maternal care guidelines’, ‘facilitators to the implementation of maternal care guidelines’, ‘midwives’ roles in implementing maternity guidelines’, ‘midwives’ views in implementing maternity guidelines’, ‘postnatal health’, ‘postnatal care services’. Boolean terms (AND, OR) were used to separate the keywords during the search. Medical Subject Headings (Mesh) terms were also included in the search. From the included studies’ list of references, we manually searched for relevant studies. The search strategy is contained in (Supplementary File 1).

#### Stage 3: Study selection and eligibility

After screening the titles from the aforementioned databases, articles with relevant study titles for this research were uploaded to the Mendeley 1.19.18 software. Search results from various electronic databases were compiled into a single Mendeley library. Studies that did not address the research question were excluded, as were duplicates of the same records. The studies’ title, abstracts and full articles of the available papers from the search were independently screened by NO and RN for quality, discrepancies and later cross-checked. The review eligibility criteria were used to create an abstract screening form with questions. The relevant studies were identified based on the inclusion and exclusion criteria developed in response to the research questions. The authors compared findings and agreed on the articles to include in the study.

### Inclusion criteria

The following inclusion criteria guided the selection:

1. Studies with no language restrictions/ published in the English language
2. Studies that focus on maternal health especially postnatal health for mothers and newborns
3. Studies published between the years 2017 to 2023
4. Studies that report the roles, views, and experiences of midwives.

### Exclusion Criteria

The following exclusion criteria were used:

1. Studies that focus on health and health services other than maternity and postnatal health
2. Studies not published in English
3. Studies that were published before January 2017
4. Studies that do not report the roles, views, and experiences of midwives.

Therefore, the study excluded other aspects of health, not maternal health and postnatal health. Also excluded are studies published before 2017. Studies were identified by searching literatures published in English as it is the commonly used language for communication in most Sub-Saharan African countries. We restricted the search to include studies published from January 2017 to June 2023 because initial searches of the literature showed that most relevant studies were conducted after 2017. Additionally, a 6-year literature search is more likely to yield a comprehensive account of current research in the area. In the selection phase, articles obtained were exported to Mendeley version 1.19.18. Titles, abstracts, and full text of articles obtained from the search process were screened against the above-listed criteria, and findings were compared. Ultimately the search yielded twenty-two articles that met the study’s inclusion criteria. These were included in the study.

#### Stage 4: Charting data

An information formation form was used to extract data from each selected paper. The information included the following: study authors, study location, year of study, study design, and methodology, as well as barriers and facilitators that influence midwives’ execution of maternal care guidelines. This process helped to summarize the valuable information for reporting the findings of the review.

#### Stage 5: Collating, summarizing, and reporting results

A theme framework is necessary for a scoping study, according to Arksey and O’Malley (2005), to offer a comprehensive account of the chosen literature.

### Quality assessment

The quality of the included studies was assessed using the Mixed Method Quality Appraisal Tool (MMAT) (Pluye *et al.*, 2009). Two independent reviewers (NO and RN) evaluated the quality of the included studies based on the following criteria: the relevance of the research question, data collection, data analysis, sampling methodology accuracy, author acknowledgement of potential biases, and conclusion. The included studies were assigned a quality score ranging from <50% (low quality), 51–75% (average quality), to 76–100% (high quality). None of the twenty-two primary studies evaluated had a quality score of <50%, indicating minimal risk of bias in the overall evidence (see Supplementary file 2).

After screening the titles and removing duplicates, this scoping review identified 164 eligible studies out of 1760 articles. Following abstract screening, 114 articles were excluded. Two reviewers selected fifty (50) articles for full-article screening, and twenty-two (22) articles were selected for in-depth data extraction and methodological quality assessment. Cohen’s kappa coefficient (κ) statistic was used to measure inter-rater agreement between reviewers. The agreement between reviewers was 81.82%, lower than the expected rate of 83.47% (Kappa statistic = −0.10 and p-value > 0.05). However, McNemar’s chi-square test revealed no significant difference in yes/no responses by reviewer (p-value > 0.05). Supplementary file 3 contains a calculation for the degree of agreement. The PRISMA-ScR (Preferred Report Items for Systematic and Meta-Analysis for Scoping Review) (Moher et. al., 2015) flow diagram was further used to select and present the studies. This is specifically tailored to provide guidance, transparency and reproducibility (Miranda *et al.*, 2023) (see supplementary file 4).

### Data analysis

Thematic content analysis of the themes to identify contextual factors was performed by NO and RN. The choice of a thematic synthesis approach was made because it offers a transparent, systematic, and flexible way to find rich and detailed data for synthesis across several studies (Braun and Clarke, 2020). As a result, the five-step technique of thematic analysis suggested by Braun and Clarke (2020) was employed to identify common motifs (Braun and Clarke, 2020). The first step was data familiarization, which required thoroughly reading all 22 articles that satisfied the inclusion criteria. Initial thematic codes were created inductively during the second step. Microsoft Word was used to create the initial line-by-line codes to aid in the creation of the descriptive and analytical themes. NO took the lead on the thematic synthesis, with RN providing iterative input. While the reviewers’ analytical themes go beyond the primary studies and produce fresh interpretive insights or explanations, the descriptive themes stay closely aligned with the primary research studies that were part of the review. The initial codes were converted into emergent themes in the third step. The themes were continuously examined in step four to capture all topics that emerged from the complete data set. Making sure the thematic analysis was thorough and consistent was the fifth step. This was accomplished by carefully examining the relationships among the various themes that were discovered to group them into a significant manner.

### Author reflexivity

To help the reader contextualize the relationship between the researchers and the research, it is crucial for researchers conducting scoping reviews to be aware of the assumptions and biases they may have that could affect the methodology of the study (Byrne, 2022). The authors were professionals with both clinical and research experience. Doing this was essential to overcoming potential biases. The application of the MMAT methodological framework guided the review strategy. MMAT makes it possible to evaluate the majority of popular study designs and methodologies. Nevertheless, the MMAT is unable to evaluate certain particular designs, such as economic and diagnostic accuracy studies. For these designs, additional critical appraisal instruments may be pertinent (Pluye *et al.*, 2009, and Moher *et al.*, 2015).

### Limitations

As with most reviews, there is the possibility that some papers could have been missed during each process. It is also possible that some papers used terminologies different from the terms used in the review. Also, only papers that were published and available on the databases searched were included in the study. Due to this, some relevant studies might have been omitted (Levac *et al.*, 2017).

## Results

A comprehensive search of the literature on the subject turned up 1760 articles, of which 22 met the inclusion and exclusion criteria and were therefore deemed pertinent to the factors impacting midwives’ implementation of maternity care recommendations. In the first stage, duplication and titles without a focus on maternity and postnatal health were removed; at this stage, 740 articles were dropped, leaving 1020 articles, and another 856 articles were removed, leaving 164 papers for evaluation. In the second stage of the review, only research from sub-Saharan Africa and a few pertinent studies from South Africa were evaluated. At this point, 50 articles remained after 114 were dropped. At the third stage, 28 articles were eliminated from consideration because they did not specifically address maternal care recommendations and midwives’ responsibilities, were not focused on postpartum health for mothers and newborns, or included other health-related topics besides maternal and postnatal health. Twenty-two pieces were ultimately chosen for theme analysis. In (supplementary file 5), the search result is depicted. The majority of the publications received (n=12; 54.5%) were reviews and discussion articles, with just a small number (n=6) of evidence-based research and reviews of recommendations for postpartum health (n=4) (18.2%). The major themes were derived from the WHO (2018), quality of care standards framework identifying the eight pillars of care across dimensions, which are: evidence-based care practices, actional information systems, functional referral systems, effective communication, respect and dignified care, emotional support, competent, motivated staff, and availability of essential physical resources (Siseho *et al.*, 2022). The articles’ findings were further grouped into two sub-themes of barriers to midwives implementing maternal care standards for postnatal health and facilitators of such implementation, with several categories arising from each of these. The themes were derived from the WHO (2018), quality care standards using the eight pillars of care across dimensions to elaborate on these concepts. This is contained in (Table 1).

**Table 1.** Factors influencing midwives’ implementation of maternal care guidelines in postnatal health (source: WHO, 2018)

NO	THEMES	BARRIERS	FACILITATORS
1	Evidenced-based care practices	limited information on core information of the guidelines Poor evidence-based care and management of complications during labour, childbirth, and the early postnatal period, according to the MCG guidelines. Poorly developed/organized PNC services Weak service availability (screening/assessment, postnatal services) delayed identification of PNC complications and treatment due to a shortage of diagnostic equipment.	increased awareness of the benefits of the guidelines Ensuring that effective evidence-based safe care is provided during labour and childbirth. provision of evidence-based safe-postnatal care for all mothers and newborns. ensuring that the physical environment of the health facility is safe for providing maternal and newborn care.
2	Actional information system	Poor health information system that limits the use of data to ensure the early, appropriate action to improve the care of every woman and newborn.	Ensuring that effective health information systems are in place to manage patient clinical records and service data
3	Functional referral system	Poor referral system of maternal and newborn condition(s) that cannot be dealt with effectively with the available resources Undefined pathways to care/referral system	Appropriate referral system and maintenance of available services to ensure continuity of care for all pregnant women, mothers, and newborns
4	Effective communication	Poor health system communication and ineffective communication among midwives, women, and their families	Provision of an effective communication system among health teams, women, and their families Adequate supervision Intersectoral collaboration between the departments of health, education, and stakeholders.
5	Respect and dignified care	Disregard from the public and other health teams Limitations in ensuring that Women and newborns receive care with respect and preservation of their dignity.	Ensuring that human rights are observed and the experience of care is dignified and respectful for every woman and newborn
6	Emotional support	Poor emotional support, burnout due to shortage of staff and retention	Ensuring midwives ’mental health and provision of emotional support through appreciation and strengthening of their capability.
7	Competent motivated Staff	increased number of unmotivated and unskilled midwives	increasing support and orientation of midwives in essential skills. Improvement in salaries and remunerations.
8	Availability of essential resources	inadequate/ limited equipment, resources, and infrastructures Limited number of midwives Lack of training for midwives on M.C.G. Financial constraints for M.C.G Insufficient dedicated government funding	Ensuring that the health facility has an appropriate physical environment, with adequate water, sanitation, and energy supplies, medicines, supplies, and equipment for routine maternal and newborn care and management of complications.

### Presentation of findings

#### Key factors to the maternal care guidelines (MCG) implementation during the postpartum phase

The key factors to MCG implementation during the postnatal period were generally agreed upon in the reviewed literature. The unavailability of critical resources, equipment, and supplies was mentioned in five articles; poor retention of competent, motivated staff was mentioned in five articles. Poor service delivery was mentioned in six articles; poor evidenced-based care practices were mentioned in five articles; poor system communication and collaboration was mentioned in four articles; ineffective functional referral system was mentioned in four articles, and poor health system accountability was mentioned in three studies. Also identified was an unstructured health information system mentioned in three articles. Similarly, (Alkema *et al.*, 2017) identified some of these factors, including a lack of appropriate policies and guidelines, poor leadership and health governance, a lack of trained personnel, and a scarcity of medical equipment, supplies, and financial resources as contributing to poor postnatal phase implementation. The characteristics of the included studies are shown in Table 2.

**Table 2.** Characteristics of included studies. From: Barriers and facilitators influencing midwives’ implementation of South Africa’s maternal care guidelines in postnatal health: a scoping review

N0	AUTHOR	YEAR, LOCATION	STUDY DESIGN	BARRIERS	FACILITATORS
1	Makina-Zimalirana *et al.*,	2022, South Africa	Systematic review Qualitative	Lack of resources (space/medical equipment/staff) Lack of stakeholders’ adoption Poor service delivery	Increased support at the management level to ensure sustainability Effective collaboration among stakeholders facility leadership for successful integration
2	Solnes Miltenburg *et al.*,	2017, South Africa	Systematic review, qualitative	Lack of stakeholder participation lack of training and orientation	Contextualizing interventions through multi-stakeholder involvement increased training and awareness
3	Ramavhoya *et al.*,	2022, South Africa	Literature Qualitative	lack of human resources poor maternal health services knowledge deficit Poor service delivery	availability of the guidelines community involvement quality assurance
4	Forbes *et al.*,	2023, Sub-Saharan Africa	Systematic review	poor infrastructures lack of skills poor awareness poor service delivery Poor retention of competent and motivated staff Ineffective referral system	provision of adequate resources Skills acquisition Knowledge and increased awareness Behavioural change regulation support correction of misconceptions increased social, and professional role and identity
5	Simona *et al.*,	2022, sub-Saharan Africa	Scoping review	The level of educational status poverty, poor media exposure, poor autonomy/ empowerment poor access to health facilities	increased awareness poverty eradication and hunger empowerment and increased capacity increased access to health facilities
6	Pettersson,	2018, Sub-Saharan Africa	Systematic review	Non-conducive environment that lowers the capacity to respond to demand poor adaptation of midwifery education to the national context and evidence-based practices difficulties being a member of a low-status profession financial struggles Disregard from the public and other health teams Lack of support and necessary backing and resources to handle challenges brain drains lack of access to maternal health poor health and death owing to HIV/AIDS	Provision of a supportive environment Improvement in midwifery education and curriculum Scaling up the midwives’ functions in PNC service delivery, especially during the postnatal stage increased awareness of the roles of midwives and the importance of the MCG provision of adequate resources and supports improved access to maternal health services Improvement in midwives’ health and living conditions emotional support and improved mental wellbeing
7	Siseho *et al.*,	2022, South Africa	Systematic review	Por capacity building Poor health system coordination Poor reporting and documentation.	Improvement in the health system and capacity Adequate reporting and documentation Improvement in system coordination and collaboration
8	Lodeke	2017 Sub-Saharan Africa	Report	Lack of skilled birth attendance at delivery including prompt resuscitation Lack of postnatal care during the immediate postnatal period Lack of newborn care	Strengthen health system actions for mothers’ and newborns’ survival and health by fostering good quality postnatal health services across the health continuum increase in essential lifesaving interventions
9	Yakubu & Salisu	2018, Sub-Saharan Africa	Systematic review	lack of inadequate assessment and triage, late treatment, •inadequate drug/ supplies, poor knowledge and usage of evidence-based treatment guidelines, insufficient clinical monitoring	Ensure universal coverage of evidence-based guidelines to ensure health equity and reduce mortality improved assessment and early triage prompt identification and treatment provision of drugs/supplies education, training, and clinical evaluation
10	Bhardwaj *et al.*,	2018, South Africa	Systematic review	Health system challenges: health financing, human resources, essential supplies, and infrastructures limited health capacity	Improvement of health infrastructures improvement of health capacity and resource Better health system capacity
11	Ajewole,	2019, South Africa	Qualitative review	Poor monitoring, evaluation, and research into MCG implementation poor multisectoral approaches limited progress in implementation	Support monitoring, evaluation, and research into maternal and newborn guideline implementation promote multisectoral approaches to make progress
12	Maaløe *et al.*,	2021, Sub-Saharan Africa	Systematic review	Poor quality of care and health quality system. Failure to adopt the standard of care Poor role definition, delegation, and supervision Poor communication and collaboration.	Improvement of the health quality system. by establishing national and subnational quality cells and defining roles at various levels. adopting standards of care. Assessing the current quality of care would help in identifying common gaps in quality care within the health system
13	Hazfiarini *et al.*,	2022, Indonesia	Qualitative review	Poor attention to MCG implementation Poor health system planning and interventions poor service delivery and retention of competent staff Unstructured health system Poor system communication and collaboration.	guidelines for implementation during the postpartum periods need to receive higher, attention owing to their huge and immediate benefits for the child and mothers’ survival and health as well as long-term potential towards national development. improvement in health planning and interventions
14	Demers *et al.*,	2017 Africa	Qualitative review	Poor coverage and equity gaps wide socioeconomic disparities Challenges of scaling up with equity may be largely related to the health system and primarily to health financing, infrastructure, and human resources (specifically, midwives).	Addressing gender issues and emphasis on equal access to health interventions/recommendations provision of overall health equity-specific in meeting the need of mother and their babies, implementing the guidelines appropriate health financing and increase in human resources improved coverage and provision of respect and dignified care
15	Behruzi *et al.*,	2017, Quebec	Case study	Quality gaps and provision of suboptimal care Poor service delivery and staff.	Improvement of maternal and newborn health services especially during the time of childbirth
16	Munabi-Babigumira *et al.*,	2019, Uganda	Qualitative study	Poor health information system Poor monitoring and evaluation of health guidelines and	Strengthening health information systems at districts and regional levels, including birth and death (and cause of death) registration and the health management information system (HMIS).
17	Alkema *et al.*,	2017, Sub-Sharan Africa	Systematic review	Poor leadership and health governance absence of suitable policies and guidelines, lack of trained personnel, •scarcity of medical equipment, supplies and •financial resources	Leadership improvement in the political, economic landscape and health system Improved health governance Development and sustenance of adequate techno-managerial capacity in the health facilities to provide strong leadership and governance to the newborn and maternal health at district and regional levels
18	Graham *et al.*,	2017, South Africa	Systematic review	Poor retention of trained, experienced, and motivated midwives Poor capacity building Poor guideline implementation, planning, and development	Provision of trained, experienced, and committed staff that could provide solid and consistent leadership for guidelines review, planning, and management of implementation and monitoring progress.
19	Nyamtema *et al.*,	2017, Sub-Saharan Africa	Systematic review	Increased maternal and newborn comorbidities example HIV, diarrhoea, pneumonia Poor service delivery Poor evidenced-based care.	Scaling up interventions during intrapartum and postpartum periods in maternal and newborn health Prioritization of interventions to address common causes of maternal and newborn morbidities
20	Kurinczuk *et al.*,	2017, UK	Systematic review	Poor data-driven decision-making Unavailability of essential resources Poor service delivery	Adequate data monitoring and maintenance of progress Improvement of skills in data understanding and translation to increase guideline implementation, set priorities and suggest policy changes
21	Zahroh *et al.*,	2022, sub-Saharan Africa	Mixed method systematic review and narrative synthesis	Limited service delivery in maternity and facility areas Poor implementation strategies and planning Unavailability of essential resources	Provision of health workers (HWs) with advanced skills (qualified nurses, midwives, and physicians) to be available permanently to provide facility-based care at primary, secondary, and tertiary (critical care) levels, including childbirth, neonatal, and general paediatric care.
22	Kinney *et al.*,	2022, South Africa	Multicase study approach	limited referral system Poor measurement and accountability gaps	Improvement of functional referral system and improvement in health system evaluation and accountability

#### Barriers to midwives’ implementation of maternal care guideline (MCG) on postnatal health

Midwives’ use of MCG in the postnatal phase is influenced by the following factors: health system challenges (health financing, resources, equipment essential supplies and drugs) (Alkema *et al.*, 2017; Daemers *et al.*, 2017, Forbes *et al.*, 2023; Lodeke, 2017; Makin-Zimaliran *et al*, 2022); poor service delivery Makina-Zimalirana *et al.*, 2022; Ramavhoya *et al.*, 2022; Hazfiarini *et al.*, 2022, Behruzi *et al.*, 2017; Nyamtema *et al.*, 2017; Forbes *et al.*, 2023); poor health information, poor media exposure, and access to health facilities (Munabi-Babigumira *et al.*, 2019; Kurinczuk *et al.*, 2017; Simona *et al.*, 2022); poor health system coordination, planning, and intervention (Graham *et al.*, 2017; Hazfiarini *et al.*, 2022, Siseho *et al.*, 2022), and poor health system governance and leadership (Alkema *et al.*, 2017). Furthermore, there is a lack of awareness, attention, and implementation of guidelines (Graham *et al.*, 2017; Pettersson, 2018; Ramavhoya *et al.*, 2022; Simona *et al.*, 2022, Yakubu, 2018); lack of trained, competent, skilled, and motivated; and poor retention of midwives (Alkema *et al.*, 2017; Ramavhoya *et al.*, 2022; Graham *et al.*, 2017; Lodeke, 2017; Hazfiarini *et al.*, 2022; Forbes *et al.*, 2023). Poor practice, evaluation, and evidenced-based triage were also identified as having an impact on guideline implementation. (Yakubu, 2018; Lodeke, 2017; Pettersson, 2018).

Other important factors influencing midwives’ implementation of the guidelines included a non-conducive environment, a lack of support, autonomy, and empowerment (Simona *et al.*, 2022; Pettersson (2018), and poor role definition, delegation, and supervision (Maaløe *et al.*, 2021). Poor health conditions, burnout, financial struggles, and disregard from public and health teams were also identified (Simona *et al.*, 2022; Pettersson, 2018), as well as a lack of training, education, orientation, and poor adaptation to midwifery education (Forbes *et al.*, 2023; Solnes Miltenburg *et al.*, 2017; Pettersson, 2018).

Furthermore, studies have identified a lack of skilled care during the immediate postnatal period, newborn care, and lack of access to maternal care services (Lodeke, 2017; Pettersson, 2018; Ramavhoya, *et al.*, 2022; Simona *et al.*, 2022); poor reporting and documentation (Siseho *et al*, 2022); and increased risk for mothers and newborns (H.I.V, pneumonia, malformations) (Nyamtema *et al.*, 2017) as significant barriers to executing the recommendations. Other identified barriers included quality gaps such as poor quality and system control, as well as accountability and measurement (Maaløe *et al.*, 2021; Behruzi *et al.*, 2017; Kinney *et al.,* 2022) were other identified barriers. Poor health system communication, collaboration, and capacity building (Hazfiarini *et al.*, 2022; Maaløe *et al.*, 2021; Siseho *et al.*, 2022; Graham *et al.*, 2017); insufficient clinical monitoring, evaluation, and research on maternal and neonatal guidelines (Ajewole, 2019; Yakubu, 2018; Munabi-Babigumira *et al.*, 2019); were among noted barriers to poor guideline implementation. Furthermore, ineffective referral systems, poor coverage, and equity gaps (Daemers *et al.*, 2017; Forbes *et al.*, 2023; Kinney *et al.*, 2022); as well as poor multisectoral approach, and a lack of stakeholder partnership and involvement (Makina-Zimalirana *et al.*, 2022; Solnes Miltenburg *et al.*, 2017; Ajewole, 2019) were identified.

#### Facilitators to midwives’ implementation of maternal care guidelines (MCG) on postnatal health

The following factors facilitate midwives’ use of the guidelines: increased awareness of the roles of midwives and the importance of the MCG (Forbes *et al.*, 2023; Lodeke, 2017); provision of adequate resources and support (Pettersson, 2018); midwives’ education, training and evaluation of functions (Yakubu & Salisu, 2018); and scaling up the midwives’ functions in PNC service delivery, particularly during the postnatal stage (Pettersson, 2018). key factors that have been shown to improve guideline execution include improvements in health system coordination, collaboration, and capacity (Siseho *et al.*, 2022), adequate reporting and documentation (Siseho *et al.*, 2022); strengthening of the health information systems (Munabi-Babigumira *et al.*, 2019), and improvement in essential lifesaving interventions to ensure optimal health for mothers and newborns (Lodeke, 2017). Additionally, appropriate health financing and increase in human resources (Daemers *et al.*, 2017); health system governance and leadership (Alkema *et al.*, 2017); functional referral system, health system evaluation, and accountability (Kimey *et al.*, 2022); as well as provision of health infrastructures, supplies, and equipment (Bhardwaj *et al.*, 2018) are amongst factors that will improve postnatal health when the maternity guidelines are implemented. Other factors include data monitoring and maintenance (Kurinczuk *et al.*, 2017); quality control (Maaløe *et al.*, 2021); planning and scaling up interventions in maternal and newborn health during intrapartum and postpartum periods (Hazfiarini *et al.*, 2022); provision of trained, experienced and motivated midwives to ensure guidelines execution (Graham *et al.*, 2017); skill improvement (Nyamtema *et al.*, 2017).

### Findings of the major themes

#### Evidenced-based services

The provision of evidenced care is an essential component of the maternal care guideline. This is important to improve maternal and newborn health conditions; however, there are limitations due to some factors, such as poor health system coordination, planning, and intervention (Graham *et al.*, 2017; Hazfiarini *et al.*, 2022, Siseho *et al.*, 2022), as well as poor health system governance and leadership (Alkema *et al.*, 2017); these challenges could be overcome (Siseho *et al.*, 2022). The majority of the reviewed literature suggested that a major obstacle to implementing guidelines was poor service delivery in the care and management of complications during labour, childbirth, and the early postnatal period (Makina-Zimalirana *et al.*, 2022; Ramavhoya *et al.*, 2022; Hazfiarini *et al.*, 2022, Behruzi *et al.*, 2017; Nyamtema *et al.*, 2017; Forbes *et al.*, 2023); this was attributed to poor awareness on core information of the guidelines and its benefits (Graham *et al.*, 2017; Pettersson, 2018; Ramavhoya *et al.*, 2022; Simona *et al.*, 2022, Yakubu, 2018); Thus the need for greater understanding of the advantages of the guidelines is one of the contributing reasons (Forbes *et al.*, 2023; Lodeke, 2017) By ensuring that efficient and effective care is given throughout labour and childbirth (Hazfiarini *et al.*, 2022; Lodeke, 2017; Behruzi *et al.*, 2017).

Some studies identified poorly developed, unorganized postnatal care services and limited service availability such as poor screening, and assessment (Maaløe *et al.*, 2021; Behruzi *et al.*, 2017; Kimey *et al.*, 2022) as limitations in executing the guidelines. This difficulty could be overcome by prompt identification and treatment as well as adequate monitoring and evaluation (Yakubu, 2018; Ajewole, 2019). Quality assurance and control (Makina-Zimalirana *et al.*, 2022; Ramavhoya *et al.*, 2022; Hazfiarini *et al.*, 2022) are important factors influencing midwives’ implementation of the guidelines. This is important to ensure early detection and treatment of PNC complications (Pettersson, 2018), as well as ensure a safe environment for providing maternal and newborn care (Lodeke, 2017; Maaløe *et al.*, 2021); however, there are still limitations due to quality gaps, such as poor quality and system control, as well as poor accountability and measurement (Maaløe *et al.*, 2021; Behruzi *et al.*, 2017; Kimey *et al.*, 2022). There is a need for more maternity guidelines monitoring, evaluation, and research. Some studies have identified insufficient clinical monitoring, evaluation, and research on maternal and neonatal guidelines as key factors limiting guideline implementation, (Ajewole, 2019; Yakubu, 2018; Munabi-Babigumira *et al.*, 2019).

#### An actional information system

Effective information system assists midwives in assessing current quality care and identifying gaps in care within the health system (Maaløe *et al.*, 2021). This may be a challenge because the health information system has limitations that limit data use to ensure early, appropriate action to improve the care of every woman and newborn (Maaløe *et al.*, 2021). Poor coverage, equity gaps, and wide socioeconomic disparities have been identified as major barriers to guideline implementation (Yakubu, 2018). Scaling-up equity challenges may be primarily related to the health system, specifically health financing, infrastructure, and human resources (Yakubu, 2018). This issue could be addressed by increasing maternal and newborn access to maternal care services, particularly in the postnatal care stage, as well as funding various health systems, increasing human resources, and improving infrastructures (Alkema *et al.*, 2017; Daemers *et al.*, 2017, Forbes *et al.*, 2023; Lodeke, 2017; Makina-Zimalirana *et al.*, 2022). Furthermore, to ensure universal coverage of evidenced-based guidelines and ensure health equity and reduce mortality, (Yakubu, 2018). Poor health information, access to health facilities, monitoring, and evaluation have all been shown to impede the delivery of quality care as recommended by guidelines. (Munabi-Babigumira *et al.*, 2019; Kurinczuk *et al.*, 2017; Simona *et al.*, 2022). When information is not properly disseminated, there is a lack of knowledge and awareness among the health team, which can lead to communication gaps and impede healthcare services; thus, there is a need for information improvement through effective monitoring and evaluation (Kurinczuk *et al.*, 2017; Simona *et al.*, 2022; Munabi-Babigumira *et al.*, 2019). Promoting the midwife’s role in ensuring that effective health information systems are in place to manage patient clinical records and service data and strengthening health information systems at districts and regional levels will help implement the guidelines and improve health conditions (Munabi-Babigumira *et al.*, 2019). Improved data comprehension and translation skills to increase guidelines implementation, set priorities, and suggest policy changes are major factors influencing midwives implementing the guidelines (Kurinczuk *et al.*, 2017); however, there are barriers due to poor data-driven decision-making during the postnatal periods, as well as insufficient data monitoring and maintenance of progress in labour and child delivery (Kurinczuk *et al.*, 2017). These obstacles impede midwives’ roles in providing effective PNC services and have an impact on mother and baby health; thus, there is room for improvement.

#### A functional referral system

Appropriate referral system and maintenance of available services to ensure continuity of care for all pregnant women, mothers, and newborns (Siseho *et al.*, 2022), are measures that aid midwives in managing postnatal complications by ensuring early identification of these for referrals; however, this may become a challenge due to limitations in undefined pathways to care, system coordination, and collaborations in the system; thus, there is need for effective referral system (Siseho *et al.*, 2022). Poor equipment, a lack of human resources, and communication breakdowns among health teams have been identified as major significant barriers to functional referrals in healthcare settings (Alkema *et al.*, 2017; Daemers *et al.*, 2017, Forbes *et al.*, 2023; Lodeke, 2017; Makin-Zimaliran *et al.*, 2022). The dearth of equipment to monitor progress during labour, birth, and postpartum periods is a challenge, as is the shortage of midwives to manage mothers and their babies, as well as communication gaps; thus, appropriate health financing and an increase in human resources are required (Daemers *et al.*, 2017). Improvement in clinical monitoring and evaluation improvement measures influencing effective system referral as recommended by the guideline (Ajewole, 2019; Yakubu, 2018); however, there are limitations due to insufficient clinical monitoring and evaluation (Ajewole, 2019; Yakubu, 2018); this impedes midwives function leading to poor guideline implementation, service delivery, and accountability (Kinney *et al.*, 2022), thus the need for improvement

#### Effective communication

An effective communication system among health teams, women, and their families is critical in maternal health care because it reduces maternal and newborn mortalities. Unfortunately, there are limitations to these in the healthcare delivery system due to poor health system communication, collaboration, and capacity building (Hazfiarini *et al.*, 2022; Maaløe *et al.*, 2021; Siseho *et al.*, 2022; Graham *et al.*, 2017). According to studies, there is a lack of awareness, attention, and implementation of guidelines (Graham *et al.*, 2017; Pettersson, 2018; Ramavhoya *et al.*, 2022; Simona *et al.*, 2022, Yakubu, 2018); this is due to communication gaps, which result in poor quality of care and health quality system; thus, there is need to raise awareness about the benefits of the guidelines. Improvements in system coordination and planning have been identified as factors influencing effective guideline implementation (Graham *et al.*, 2017; Hazfiarini *et al.*, 2022, Siseho *et al.*, 2022); however, barriers to effective guideline implementation exist due to poor health system coordination, planning, and intervention (Graham *et al.*, 2017; Hazfiarini *et al.*, 2022, Siseho *et al.*, 2022). This may impede healthcare system communication and midwives’ functions in implementing the guidelines, leaving room for improvement. Some noted barriers to guideline implementation include poor health system communication and ineffective communication among health teams, women, and their families (Maaløe *et al.*, 2021; Forbes *et al.*, 2023). According to Hazfiarini *et al.*, 2022; Maaløe *et al.*, 2021; Forbes *et al.*, 2023, when there are gaps in communication among health teams and patients, there are decreased health outcomes and suboptimal care, necessitating improved communication and information among members of the health team, women and their families (Simona *et al.*, 2022). Some identified factors to promote guideline implementation include effective delegation and supervision. This is significant because it improves communication, clinical monitoring, and evaluation, as recommended by the guidelines (Ajewole, 2019; Yakubu, 2018; Munabi-Babigumira *et al.*, 2019); however, there are limitations due to the failure to implement the standard of care and poor role definition, delegation, and supervision (Maaløe *et al.*, 2021; Forbes *et al.*, 2023) leaving room for improvement.

## Respect and dignified care

In implementing guidelines, factors to consider include ensuring that human rights are observed and that the experience of care is dignified and respectful for every woman and newborn, as well as respecting the well-being of the health workers, particularly midwives. Both parties’ rights must be valued for a functional health system, though there are limitations due to misconceptions and communication gaps (Pettersson, 2018; Hazfiarini *et al.*, 2022). Midwives frequently face challenges that impede their roles in effectively executing the guidelines due to disregard from the public and other health members, as well as limitations in ensuring that women and newborns receive adequate care with respect (Lodeke, 2017; Pettersson, 2018). Hence, there is a need to appreciate and support midwives in their roles so they can render the required dignified care to patients at all times (Pettersson, 2018). Furthermore, studies have identified a lack of skilled care during the immediate postnatal period, newborn care, and lack of access to maternal care services as some barriers that affect the delivery of dignified care to patients; thus, there is a need to employ and retain skilled birth attendants, particularly in the maternity units and improve access to maternal services. (Pettersson, 2018; Ramavhoya, *et al.*, 2022; Simona *et al.*, 2022; Lodeke, 2017).

### Emotional support

The mental health and emotional well-being of every skilled worker is an essential factor to consider when it comes to providing quality maternal and neonatal care. Personal challenges, health conditions, anxiety, stress, burnout, an unconducive environment, and lack of support are among the many challenges that health workers face, which often conflict with their roles in the delivery of care and the execution of the guideline (Graham *et al.*, 2017; Pettersson, 2018; Ramavhoya *et al.*, 2022; Simona *et al.*, 2022, Yakubu, 2018). These and other factors may make implementing the guideline effectively and providing quality care difficult. According to studies, poor emotional support, burnout due to staff shortages, and retention are key barriers to guideline implementation (Pettersson, 2018; Ramavhoya *et al.*, 2022; Simona *et al.*, 2022, Yakubu, 2018). As a result, these areas need improvement by ensuring midwives’ mental health and providing emotional support through appreciation and strengthening of their capability.

### Competent, motivated staffs

The availability of competent, motivated midwives is critical to the delivery of effective postnatal services using the MCG because they are vital members of the health team. When staff are motivated, they function optimally and standards of care are increased; however, there are limitations to these due to a lack of support, a non-conducive environment, autonomy, and empowerment (Simona *et al.*, 2022; Pettersson, 2018). Thus, there is a need for intervention in these critical areas. A lack of trained, skilled, and motivated midwives, as well as poor retention of midwives have been identified as significant barriers to guidelines implementation in studies (Alkema *et al.*, 2017; Ramavohov *et al.*, 2022; Graham *et al.*, 2017; Lodeke, 2017; Hazfiarini *et al.*, 2022; Forbes *et al.*, 2023). Thus, for midwives to be competent in providing effective postnatal care, they must be trained and well-orientated in essential skills. Employing and retaining more midwives in the maternity units is also necessary. Improved salaries and remunerations, as well as increased support and orientation of midwives in essential skills, are major measures that would help motivate them to function in their respective roles (Lodeke, 2017, Pettersson, 2018). However, studies have shown that midwives frequently face challenges such as poor health conditions, burnout, financial difficulties, and disregard from the public and health team were also identified (Simona *et al.*, 2022; Pettersson, 2018). Poor role definition, delegation, and supervision were identified as factors that impede the midwife’s responsibilities (Maaløe *et al.*, 2021). This could be due to a lack of training, education, orientation, or poor adaptation to midwifery education (Forbes *et al.*, 2023; Solnes Miltenburg *et al.*, 2017; Pettersson, 2018). As a result, there is a need for improvement in midwifery education, training, and clinical evaluation (Yakubu, 2018). Poor practice, evaluation, and evidenced-based triage have all been identified as factors influencing guideline implementation. (Yakubu, 2018; Lodeke, 2017; Pettersson, 2018; Ramavhoya, *et al.*, 2022; Simona *et al.*, 2022).

### Availability of essential resources

The health care system can only function effectively when that are available resources in terms of people, supplies, and equipment, but studies have shown that some health system challenges impede the implementation of guidelines, such as health financing, resources, equipment essential supplies, and drugs (Alkema *et al.*, 2017; Daemers *et al.*, 2017, Forbes *et al.*, 2023; Lodeke, 2017; Makin-Zimaliran *et al.*, 2022). Providing an appropriate physical environment with adequate water, sanitation, energy, supplies, medicines, and equipment for routine maternal and newborn care as well as management of complications, are measures that promote quality care and optimal health conditions (Daemers *et al.*, 2017; Forbes *et al.*, 2023; Lodeke, 2017; Makin-Zimaliran *et al.*, 2022). Studies have however shown that these have limitations due to poor health. Some identified factors that influence the availability of resources include health system coordination, planning, and intervention (Graham *et al.*, 2017; Hazfiarini *et al.*, 2022, Siseho *et al.*, 2022), as well as health system governance and leadership (Alkema *et al.*, 2017). As a result, there is a need for improvements in health finance, infrastructures, resources, equipment, essential supplies, and drugs (Alkema *et al.*, 2017; Daemers *et al.*, 2017, Forbes *et al.*, 2023; Lodeke, 2017; Makin-Zimaliran *et al.*, 2022).

## Discussion

Globally, postnatal coverage and programme levels are still at their lowest across the care continuum, and Africa is no exception (World Health Organization., 2017), where at least one hundred and twenty-five thousand women and eight hundred and seventy-thousand newborns die each year (World Health Organization, 2017). The first few weeks are the most dangerous for both the mother and her child (Abota, Tadele & Atenafu, 2018). As a result, mothers and newborns should be closely monitored and evaluated throughout this stage (Sayinzoga *et al.*, 2018). According to the Saving Moms report (NDoH, 2017:3) and (Moodley *et al.*, 2018), 92% of pregnancies get at least one antenatal contact, and 91% of births take place in a facility with a trained birth attendant present. When compared to other sub-Saharan African countries, South Africa has strong coverage of important maternal health interventions. Even though this might be a sign of immediate postnatal care. It does not demonstrate how effectively the patient was cared for. This could be a factor in the gradual decline in maternal death rates in South Africa (NDoH, 2017; Moodley, Fawcus & Pattison, 2018).

There have been few studies on maternal guideline implementation (Bradshaw and Dorrington, 2017; Pattison *et al.*, 2018; Ramavhoya, *et al.*, 2020). The South African National Department of Health, (2018) and the National Committee for Confidential Enquiry into Maternal Deaths (2018) did not state the efficiency of implementation or describe how the guideline could be implemented. Despite this, the South African Department of Health 2015; and Elgonda *et al.*, 2018) used the national maternity care policies as a framework to describe some factors in the maternal care guideline, which include the provision of obstetric care and skilled midwifery care, appropriate referral, regionalized care, capacity building, ongoing service audits, and research (Ojuri-King *et al.*, 2018; Moodley, Fawcus, & Pattison, 2018). As it stands, the effectiveness of MCG’s implementation, however, is currently unknown (South African National Department of Health, 2018; National Committee for Confidential Enquiry into Maternal Deaths, 2019: 5; Moodley, Fawcus, & Pattison, 2018:10).

According to Dorrington, Bradshaw, and Laubscher (2019), there is a need for appropriate interventions, such as prompt diagnosis, appropriate management of maternal and neonatal complications, including the referral process to higher levels of care, and a more direct approach so that medical professional particularly midwives can more easily adopt the management plan (South African Department of Health, 2017;19; National Committee for Confidential Enquiry into Maternal Deaths, 2019). Because of this, the effectiveness of the guidelines depends on how effectively health professionals execute them as well as how the health system drafts policies based on the information and then adapts them to take into account particular circumstances (South African Department of Health, 2018: 21; Wibbelink *et al.*, 2022). Due to the limitations of applying these guidelines and the ineffectiveness of these factors, they have not been thoroughly investigated, even though they may have changed over time (South African National Department of Health, 2018; Saving Mothers Report, 2017).

The South African Maternal Care Guidelines provided a plan for midwives’ role in establishing successful postpartum care for mothers and newborns (South African Department of Health, 2017). The mother care recommendations are intended to be used at clinics, community centres, and district hospitals that provide maternal health care services (South African Department of Health, 2017;17). It is assumed that for the MCG to be effective, healthcare professionals should have a fundamental understanding of how to care for expectant mothers and that the health system would incorporate the crucial data in the guidelines to adhere to its protocol for client management and care delivery (South African Department of Health, 2017;18) (Walker *et al.*, 2019). This presents a challenge because it is hard to coordinate postpartum care (PNC) for mothers and their infants and to put the recommendations into practice (Warren *et al.*, 2017) due to poor health system coordination, planning, and intervention (Graham *et al.*, 2017; Hazfiarini *et al.*, 2022, Siseho *et al.*, 2022), as well as poor health system governance and leadership (Alkema *et al.*, 2017); these challenges could be overcome (Siseho *et al.*, 2022).

Without midwives, the healthcare system would be incomplete. They provide health services to women and their infants per the maternity care recommendations (National Committee for Confidential Enquiry into Maternal Deaths, 2019; South African Department of Health, 2015). Several factors, however, severely influence how they play their roles in implementing the maternal guidelines into practice during the postpartum periods. The National Committee for Confidential Enquiry into Maternal Deaths (NCCEMD), 2018 lists a few of these: a lack of comprehensive information about maternal care guidelines, a shortage and low retention of midwives in the postnatal wards, insufficient orientation and training of midwives, unmotivated midwives, scarce resources and equipment, inadequate capacity building, and funds (NCCEMD, 2018; Mulaudzi *et al.*, 2017; Santhoshkumari and Sharmil, 2022).

According to the findings from studies, midwives’ implementation of maternity guidelines remains poor, and evidenced-based postnatal care is scarce (Ramavhoya *et al.*, 2020; Healy *et al.*, 2017). Collecting studies of maternal and newborn care from around the world provides collaborative evidence of the barriers to the delivery of quality PNC services, such as a paucity of financial and human resources, which is a major factor in any functional health system (Alkema *et al.*, 2017; Daemers *et al.*, 2017, Forbes *et al.*, 2023; Lodeke, 2017; Makina-Zimalirana *et al.*, 2022). The majority of the studies identified a shortage of competent midwives as a major barrier to the delivery of PNC services, which is exacerbated by socioeconomic inequalities (Graham *et al.*, 2017; Lodeke, 2017; Hazfiarini *et al.*, 2022; Forbes *et al.*, 2023; Pettersson, 2018). One of the contributing factors is the need to empower trained midwives, with the limited training opportunities available impeding their development and workforce (Simona *et al.*, 2022; Pettersson, 2018). Furthermore, some studies identified a major barrier to guideline implementation: lack of professional midwives available to work in low-resource settings (Forbes *et al.*, 2023; Solnes Miltenburg *et al.*, 2017; Pettersson, 2018). According to some studies, the available midwives were not aware of the guideline’s core information and generally tended to provide services to newborns and mothers using the old approaches, resulting in frequent overburdening (Graham *et al.*, 2017; Pettersson, 2018; Ramavhoya *et al.*, 2022; Simona *et al.*, 2022, & Yakubu, 2018). Despite this, some midwives were opposed to rule changes and instead treated expectant patients as they had been taught throughout their foundation midwifery training (Ramavhoya *et al.*, 2020; Healy *et al.*, 2017). It is not surpassing that most of the studies recommended pre-service and in-service training for midwives and skilled birth attendants at provincial and district levels to improve health outcomes (Yakubu and Salisu, 2018; Pettersson, 2018).

According to Pattinson *et al.*, (2018), the results of the training at different levels of care regarding the implementation of the guideline showed that the training might not be sufficiently intensive and that it would be preferable for the staff members involved in maternity care to be trained at different levels of care (Pattinson *et al.*, 2018), it was suggested that the implementation of the guidelines include proper training of physicians, midwives, and other staff members involved in the care of women at the provincial level. As a result, they would have the skills and knowledge necessary to manage maternal and newborn complications (Pattinson *et al.*, 2018, NDoH, 2017:55). However, it is important to note that midwives are frontline health care professionals who treat mothers and their infants per the maternal guidelines; however, it is conceivable to find that the majority of them lack the necessary knowledge or training to provide women and newborns with the best care possible (Montgomery and Laury, 2019; Moodley, Fawcus & Pattison, 2018). Therefore, for safety and successful delivery, the midwives’ knowledge, competency, and abilities are crucial. To provide quality postnatal care, midwife education, and orientation may be a good option.

The creation and prompt management of typical, high-risk pregnancies as advised by the guidelines, as well as the provision of high-quality postnatal care, facilitate expert midwifery and obstetric care (WHO, 2018:4; Dorrington, Bradshaw & Laubscher, 2019; Chhetri, Shah, & Rajbansh, 2020). High-quality postnatal care is provided by the framework for proficient obstetric and midwifery care. To give women the best postpartum care possible, midwives and doctors should be prepared (Chhetri, Shah, & Rajbansh, 2020). Nevertheless, due to a shortage of personnel, inadequate retaining of midwives in the maternal and postnatal units, and a dearth of knowledge of fundamental information regarding maternal care, they are still limited in their ability to provide the needed adept obstetric and midwifery care (Dorrington, Bradshaw & Laubscher, 2019; WHO, 2018; Ngunyulu *et al.*, 2018; Pettersson, 2018).

A review of South Africa’s maternity care recommendations found that medical personnel are more knowledgeable, skilled, enthusiastic, and motivated when providing maternal care (Mulaudzi *et al.*, 2017). Results demonstrate that, at levels of care where this strategy has been implemented, motivated healthcare professionals, in particular midwives, can provide more maternal care services and better-quality care at levels of care with a significant reduction in maternal mortality rate to fifty-percent (Pattinson *et al.*, 2018) (South African National Department of Health, 2018). However, these are constrained by a non-conducive environment, a lack of support, autonomy, and empowerment (Simona *et al.*, 2022; Pettersson, 2018), and poor role definition, delegation, and supervision (Maaløe *et al.*, 2021). Additionally, identified were poor health conditions, burnout, financial difficulties, and disregard from the public and health team (Simona *et al.*, 2022; Pettersson, 2018). It is astounding that studies suggest raising salaries and remunerations, improving health, hiring, and providing more support to midwives are essential (Simona *et al.*, 2022; Pettersson, 2018).

The recommendation lists regionalized care and an appropriate referral system as contributing factors. The district is the basic unit of the health care region, and it is represented by the district hospital as well as some health centres. The three components of the district healthcare pyramids are the community, health centre, and district hospital (South African Department of Health, 2015;18). The three layers must cooperate to deliver services effectively and efficiently while reducing health costs (South African Department of Health, 2015;18). In addition, to provide women with the best care possible, regionalized care and a coordinated referral system are required (South African Department of Health, 2015:16; Malwela *et al.*, 2022). Nonetheless, this is limited by ineffective collaboration and communication within the healthcare systems (Saving Mothers Report, 2018). As a result, there is a need for increased capacity building, collaboration, and communication within the health system (Hazfiarini *et al.*, 2022; Maaløe *et al.*, 2021; Siseho *et al.*, 2022; Graham *et al.*, 2017). Information sharing among the health team, women, and their families has also increased (Simona *et al.*, 2022).

A strategy for overcoming barriers to universal health care coverage and information sharing among health teams is the incorporation of mobile health technology (mobile health or mHealth) unto the health system. An example of the use of the said mHealth is the Mom-Connect Program (Kabonga *et al.*, 2018). South Africa has made significant progress in improving the health information system by establishing a strategic plan to lower infant and maternal mortality and increasing the utilization of postnatal service to meet the health outcomes codified under the goals of sustainable development (Kabonga *et al.*, 2018), which include addressing the maternal child health (MCH) service delivery challenges. To support MCH, the National Department of Health of South Africa introduced the Mom Connect programme in August 2014 (NDoH, 2016). It was determined that mobile health or mHealth, is an efficient way to increase the adoption of MCH and PNC services and promote guideline implementation. Even though Mom Connect is widely used, there are still constraints to its uptake, as little is known about how, why, for whom, and under what health system conditions it improves (Kabonga *et al.*, 2018).

Key metrics include capacity expansion, efficient staff, resource management, and financial planning. Midwives and other healthcare professionals who provide maternity and postnatal care depend on the district health system functioning effectively, which is why this is important. Inadequate management, according to Vogel *et al.* (2019), is a major issue with the provision of health services (Kruk *et al.*, 2017). The health system is undoubtedly impacted by inadequate monitoring and supervision (Ramavhoya *et al.*, 2020), which hinders the recommendations’ implementation (Saving Mothers Report, 2018; 4; (Moran *et al.*, 2017). Examining audit services and practice is crucial to improving current PNC services, developing new treatments as necessary, and implementing the guidelines (Cornthwaite *et al.*, 2017; Ramavhoya *et al.*, 2020). Medical and nursing audits are necessary at many levels of the healthcare system (du Plessis, van Rooyen, and Ten Ham-Baloyi, 2021). This will encourage the use of the suggestions and uphold the high standards for maternity and postnatal care (Siseho *et al.*, 2022). However, studies have shown that there are gaps in reporting, monitoring, and evaluation processes for health systems (Lotto, 2017; Jolivet *et al.*, 2018; Ramavhoya *et al.*, 2020), which have affected the roles of midwives in implementing the recommendations, particularly during the postnatal periods (Saving Mothers Report, 2018).

Significant research on the application of maternal guidelines for postnatal health is helpful in the development of interventions and maternal health policies (Fawcus and Moodley, 2018). These initiatives include evaluating and operationalizing postnatal care services (Pintye *et al.*, 2018), improving postnatal health by involving stakeholders’ involvement (Bohren *et al.*, 2018;Sayinzoga *et al.*, 2018), and creating postpartum care plans that are affordable (Mulaudzi *et al.*, 2017; Hamilton *et al.*, 2018). The implementation of these actions is still constrained due to a lack of cooperation and partnership (Pattinson *et al.*, 2018. Therefore, it is crucial to promote midwives’ roles in creating and implementing appropriate measures. Increasing awareness of the fundamental information regarding maternal care guidelines, ensuring the evidenced-based practice of the guidelines in their role during the postnatal periods, orienting and training midwives about the guidelines, scaling up midwives and midwifery education programmes, motivating midwives to take effective actions in the postnatal periods, building capacity and preparedness during emergencies to bridge gaps in the service delivery system are some measures (Bingham *et al.*, 2018; Walker *et al.*, 2019; Ngunyulu *et al.*, 2018). In addition, promoting their roles in the provision of high-quality postnatal care also depends on a supportive environment, the availability of resources and equipment, the expansion of human resources through the hiring and retention of more midwives, the development of skills, ongoing education, effective leadership, supportive policies, cooperation with stakeholders, and an efficient referral system (Jordan *et al.*, 2018; Kumbiley *et al.*, 2022; Pettersson, 2018; Cornthwaite *et al.*, 2017; Ramavhoya *et al.*, 2020).

## Conclusion

The study showed a complex set of factors influencing midwives’ implementation of maternal care guidelines in postnatal health. Several major barriers and facilitators were discussed. This review lays the foundations for creating a strong postnatal care system in South Africa. The relative success of implementing maternal care guidelines in South Africa depends on health service administrators, who should improve midwives’ roles by providing a supportive environment, an adequate supply of supplies and equipment, and human capital expansion through hiring and retaining additional midwives. Health service governance is responsible for collaborating with midwives to improve the facilitators identified as impeding their ability to implement maternal care guidelines in South Africa and to reduce barriers. Maternal and infant outcomes will improve if midwives are supported in providing safe and high-quality care that adheres to maternal health guidelines.

## Supporting information

Okeke and Ngunyulu supplementary material 1 Okeke and Ngunyulu supplementary material

Okeke and Ngunyulu supplementary material 2 Okeke and Ngunyulu supplementary material

Okeke and Ngunyulu supplementary material 3 Okeke and Ngunyulu supplementary material

Okeke and Ngunyulu supplementary material 4 Okeke and Ngunyulu supplementary material

Okeke and Ngunyulu supplementary material 5 Okeke and Ngunyulu supplementary material

## Data Availability

All data generated or analyzed during this study are included in this published article and its supplementary information files.

## Supplementary material

The supplementary material for this article can be found at https://doi.org/10.1017/S1463423625000015
