# Supplementary material for: Barriers and facilitators influencing midwives’ implementation of South Africa’s maternal care guidelines in postnatal health: a scoping review
Source: Prim Health Care Res Dev. 2025 Feb 28;26:e16. doi: 10.1017/S1463423625000015 (PMC11883790; doi:10.1017/S1463423625000015)
Supplement: Okeke and Ngunyulu supplementary material 1 — Okeke and Ngunyulu supplementary material [file S1463423625000015sup001.docx]

**Supplementary file 1**

**Appendix 1.** Search strategy.

("Maternal care guidelines implementation globally "[MeSH Terms] OR ("Maternal guidelines implementation in sub-Saharan Africa "[All Fields] AND "maternal care guidelines"[MeSH Terms]) AND “maternity guidelines"[All Fields] AND “Guidelines "[All Fields] AND “Maternal afterbirth care” "[All Fields] AND "facilitators to the implementation of maternal care guidelines "[All Fields]) OR “barriers to implementation of maternal care guidelines All Fields] OR ("Barriers"[All Fields] AND "Facilitators"[All Fields]) OR "midwives’ roles in implementing maternal care guidelines"[All Fields]) AND ("postnatal health"[Subheading] OR "midwives functions in implementing guidelines"[All Fields] OR "midwives views in implementing guidelines"[All Fields] OR "post-natal phase"[MeSH Terms]) AND "post-natal health"[MeSH Terms]) AND "post-natal care services"[MeSH Terms]) AND “South Africa"[All Fields]) OR ("post-natal care to mothers and babies"[MeSH Terms] OR ("after birth care"[All Fields] AND "health"[All Fields] AND "postnatal care"[All Fields]) OR "delivery of postnatal care"[All Fields] OR ("maternal health"[All Fields] AND "neonatal health"[All Fields] AND "system"[All Fields]) OR" Midwifery care"[MeSH Terms]) AND "health care system"[All Fields]) AND ("2017/01/01"[PDat] : "2023/06/11"[PDat] AND "hospital"[MeSH Terms]).
