# Supplementary material for: Barriers and facilitators influencing midwives’ implementation of South Africa’s maternal care guidelines in postnatal health: a scoping review
Source: Prim Health Care Res Dev. 2025 Feb 28;26:e16. doi: 10.1017/S1463423625000015 (PMC11883790; doi:10.1017/S1463423625000015)
Supplement: Okeke and Ngunyulu supplementary material 3 — Okeke and Ngunyulu supplementary material [file S1463423625000015sup003.docx]

**Supplementary file 3**

**Barriers and Facilitators Influencing Midwives’ Implementation of South Africa’s Maternal Care Guidelines in Postnatal Health: a systematic scoping review**

**Appendix 3**: Article Screening

|  | Author and Year | Reviewer 1: Response | Reviewer 2: Response |
| --- | --- | --- | --- |
| 1 | Makina-Zimalirana et al., 2022 | 1 | 1 |
| 2 | Solnes Miltenburg et al., 2017 | 1 | 1 |
| 3 | Ramayhoya etal., 2022 | 1 | 1 |
| 4 | Forbes et al., 2023 | 1 | 1 |
| 5 | Simona et al., 2022 | 1 | 1 |
| 6 | Petterson, 2018 | 1 | 1 |
| 7 | Siseho et al., 2022 | 1 | 1 |
| 8 | Lodeka, 2017 | 1 | 0 |
| 9 | Yakubu and Salisu, 2018 | 1 | 1 |
| 10  11 | Bhardwaj et al., 2018  Ajewole 2019 | 1  1 | 1  1 |
| 12  13 | Maaløe et al., 2021  Hazfiarini et al., 2022 | 1  1 | 1  1 |
| 14  15  16  17  18  19  20  21  22 | Demers et al 2017  Behruzi et al., 2017  Munabi-Babigumira etal., 2019  Alkema et al., 2017  Graham et al., 2017  Nyamtema et al, 2017  Kurinczuk et al., 2017  Zahroh et al., 2022  Kinney et al, 2022 | 1  0  1  1  1  1  1  0  1 | 1  1  1  1  1  0  1  1  1 |

CALCULATIONS FOR DEGREE OF AGREEMENT USING STATA 13

RESULTS INTERPRETATION

Analysis of the results of full article screening show that there was 81.82% agreement versus 83.47% expected by chance which constitutes a considerably poor agreement between screeners (Kappa statistic = - 0.10 and p-value >0.05). However, the McNemar's chi-square statistic suggests that there is not a statistically significant difference in the proportions of yes/no answers by reviewer with p-value >0.05.
