# Supplementary material for: Barriers and facilitators influencing midwives’ implementation of South Africa’s maternal care guidelines in postnatal health: a scoping review
Source: Prim Health Care Res Dev. 2025 Feb 28;26:e16. doi: 10.1017/S1463423625000015 (PMC11883790; doi:10.1017/S1463423625000015)

**Supplementary file 4**

**Appendix 4:** PRISMA flow diagram demonstrating the selection and screening of studies (source: Moher et al., 2015)


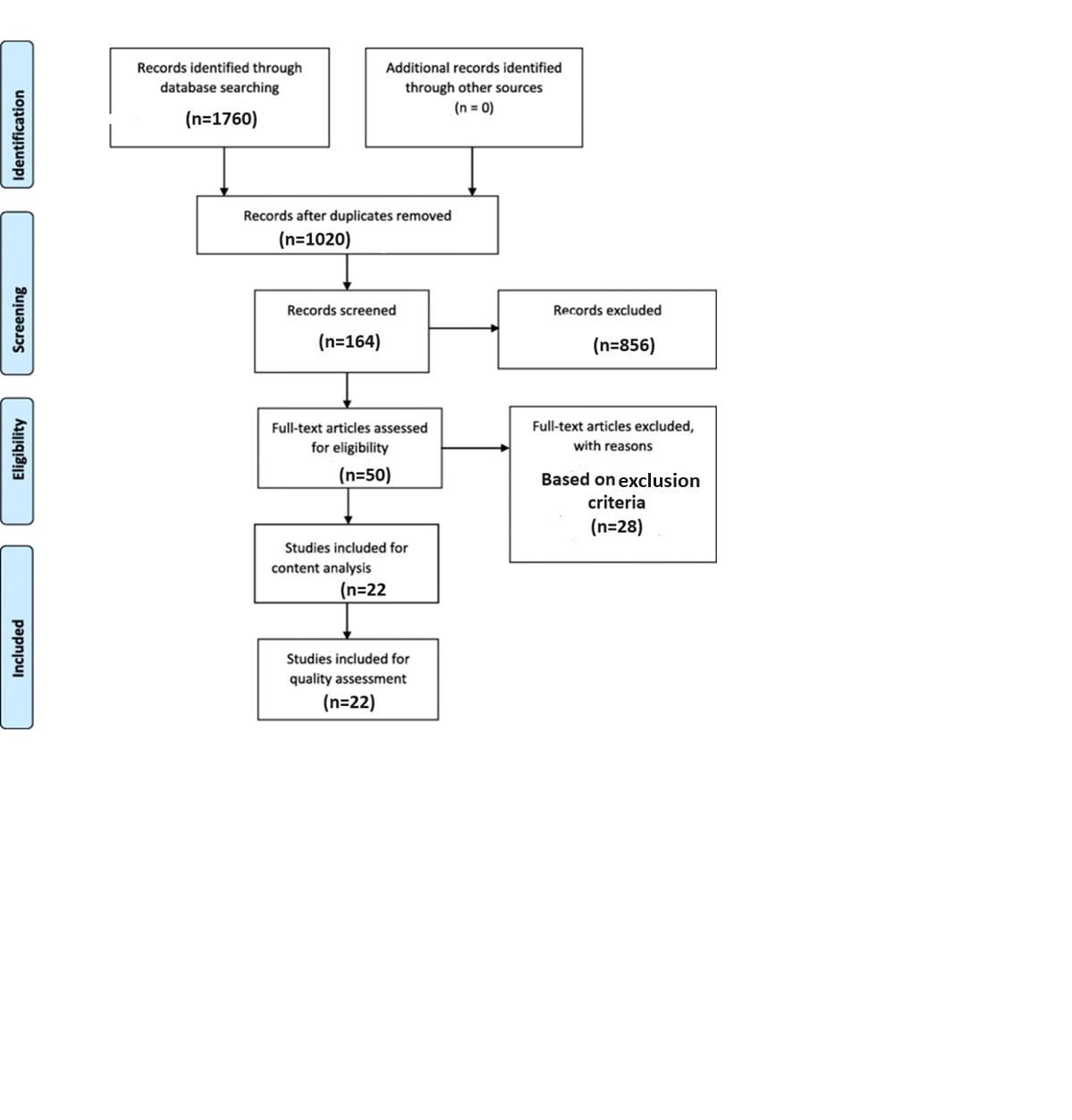

Supplement: Okeke and Ngunyulu supplementary material 4 — Okeke and Ngunyulu supplementary material [file S1463423625000015sup004.docx]
