# Supplementary figures and images for: Barriers and facilitators influencing midwives’ implementation of South Africa’s maternal care guidelines in postnatal health: a scoping review
Source: Prim Health Care Res Dev. 2025 Feb 28;26:e16. doi: 10.1017/S1463423625000015 (PMC11883790; doi:10.1017/S1463423625000015)

**Supplementary file 5**

**Appendix 5:** Search results


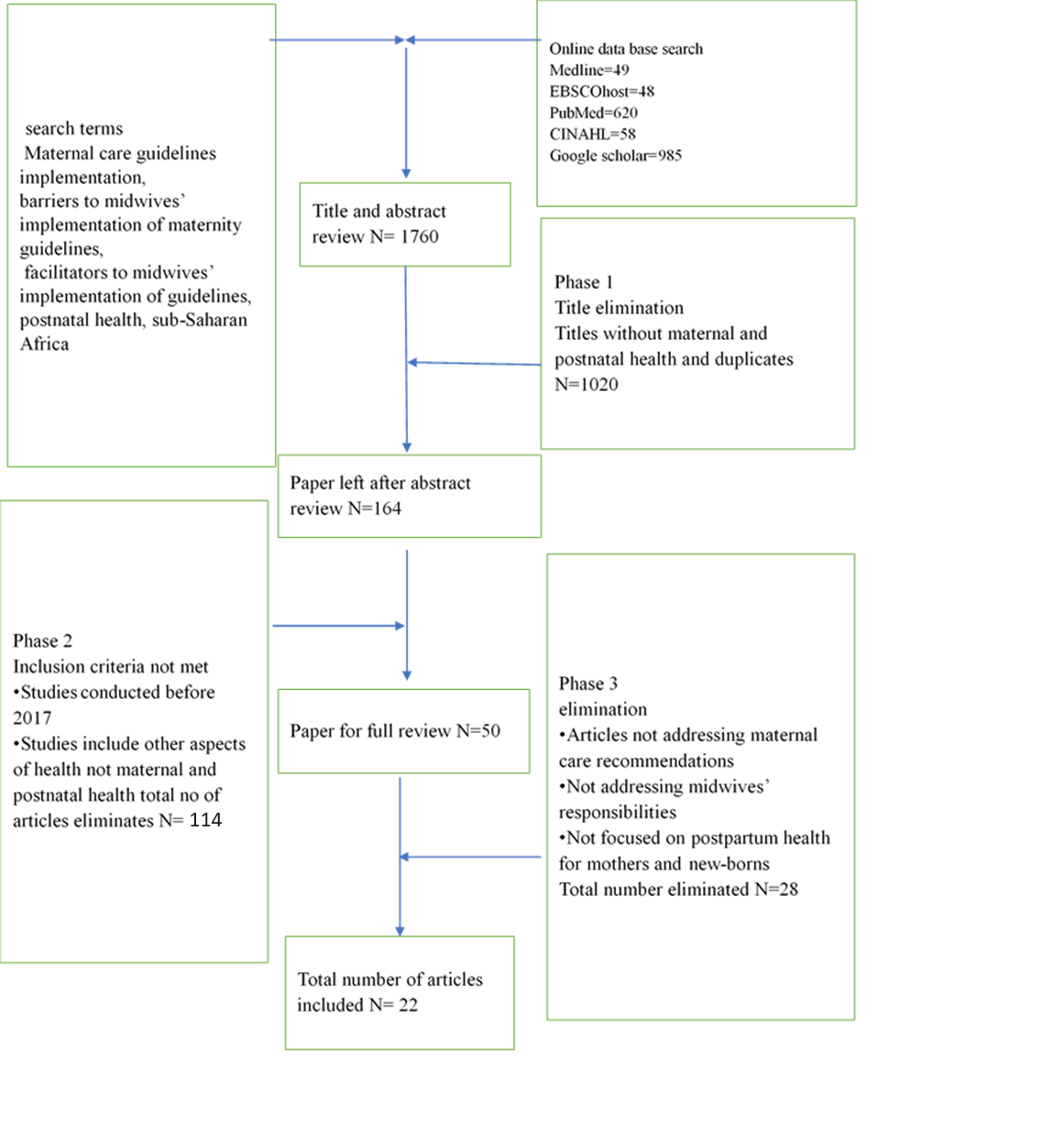

Supplement: Okeke and Ngunyulu supplementary material 5 — Okeke and Ngunyulu supplementary material [file S1463423625000015sup005.docx]
